# Severe Drug-Induced Liver Injury from Combination Encorafenib/Binimetinib

**DOI:** 10.1155/2019/3051945

**Published:** 2019-10-07

**Authors:** Nicholas Gravbrot, Srinath Sundararajan

**Affiliations:** Division of Hematology-Oncology, Department of Medicine, University of Arizona Cancer Center, Tucson, AZ, USA

## Abstract

Encorafenib/binimetinib is a new combination BRAF/MEK inhibitor used in the treatment of advanced or metastatic BRAFV600-mutant melanoma. Though generally tolerated well, mild to moderate aminotransferase elevations are common. However, significant liver injury has not been demonstrated in the literature. Here, we report the first case of severe hepatic injury associated with encorafenib/binimetinib in a 58-year-old gentleman requiring admission and extensive workup. He was successfully treated by withdrawing the combination therapy, and liver function returned to normal range.

## 1. Introduction

Encorafenib (Braftovi™, LGX818; Array BioPharma, Boulder, CO, USA) and binimetinib (Mektovi®, MEK162; Array BioPharma) are novel therapies employed in the treatment of metastatic melanoma. Both selectively inhibit distinct steps in the MAP kinase pathway (RAS/RAF/MEK/ERK), preventing tumor cell proliferation [1, 2]. Specifically, encorafenib is an ATP-competitive BRAF inhibitor (BRAFi) with longer dissociation half-life, whereas binimetinib is a non-ATP-competitive MEK1 and MEK2 inhibitor (MEKi). In a recent phase III trial (COLUMBUS) comparing combination encorafenib/binimetinib to encorafenib monotherapy and vemurafenib monotherapy, combination encorafenib/binimetinib was shown to be superior to both monotherapies in the treatment of BRAF-mutant metastatic melanoma, with improved progression-free survival (PFS), overall survival (OS), and adverse effect (AE) profile [3]. In response, combination encorafenib/binimetinib received approval from the Food and Drug Administration in June 2018 for the treatment of advanced, unresectable, or metastatic melanoma with BRAFV600 mutations.

Though typically well tolerated, encorafenib/binimetinib is associated with several potential side effects. When present, AEs related to MEK inhibition, as determined by previous phase I and II studies evaluating MEK inhibition monotherapy, predominate [4–7]. These include acneiform rash, retinal toxicity, gastrointestinal (GI) symptoms (nausea, diarrhea), and elevated creatine kinase [5–7]. Other common AEs consist of arthralgia, pruritis, hyperkeratosis, and anorexia [3, 4]. While rare (3-6% of patients), increased aspartate aminotransferase (AST) and alanine aminotransferases (ALT) levels have been reported, of which 2-5% represent grade 3 toxicity.

Herein, we describe the case of a 58-year-old male who developed grade 4 AST/ALT elevations with associated acute kidney injury shortly after initiating encorafenib/binimetinib therapy. To our knowledge, no other cases of grade 4 liver toxicity related to encorafenib/binimetinib have been reported in the literature.

## 2. Case Presentation

A 58-year-old gentleman with history of BRAF-mutant metastatic melanoma that had initially progressed after 20 months of combination dabrafenib/trametinib (BRAFi/MEKi) and again after palliative radiotherapy and three months of nivolumab (PD1 inhibitor) was started on combination encorafenib/binimetinib in January 2019. Pertinent medical history included hypercholesterolemia (on simvastatin 40 mg/day) and hypertension (on hydrochlorothiazide 25 mg/day and lisinopril 40 mg/day). He had no history of liver or kidney disease.

When encorafenib/binimetinib was initiated, the patient was essentially asymptomatic. Comprehensive metabolic panel (CMP) was unremarkable; baseline AST and ALT were 22 and 25 IU/L, respectively; creatinine was 1.23 mg/dL; and blood urea nitrogen (BUN) was 21 mg/dL. Repeat labs after the first month of treatment were similar. At a routine office visit following his second month of treatment, he reported a three-day history of fatigue, fever, and chills. AST and ALT were found to be markedly elevated, measured at 671 and 1,251 IU/L, respectively. Total bilirubin and alkaline phosphatase were within normal limits. Creatine was 2.32 mg/dL; BUN was 55 mg/dL; and glomerular filtration rate (GFR) was 49 mL/min/1.73 m^2^. Treatment was withheld, and the patient was later admitted for workup of his abnormal laboratory values due to persistent worsening of his liver function tests (LFTs) over the next two days.

On admission, hepatology was consulted to assist with the diagnostic workup. Evaluation consisted of serial CMPs, complete blood counts (CBCs), hepatitis panel, human herpesvirus panel (HSV-1, HSV-2, and VZV), autoimmune markers (antismooth muscle antibody, antimitochondrial antibody), ceruloplasmin, coagulation studies, and magnetic resonance imaging (MRI) of the abdomen with and without contrast. CMPs revealed persistent elevation of AST and ALT despite discontinuing treatment, reaching peaks of 950 and 1,638 IU/L during the course of the hospital stay. Total bilirubin and alkaline phosphatase remained within normal limits. Creatinine, BUN, and GFR gradually returned to normal with hydration after two days (0.99 mg/dL, 17 mg/dL, and 84 mL/min/1.73 m^2^, respectively). CBCs revealed normocytic anemia (hemoglobin 11.8 g/dL, MCV 86 fL, and normal iron studies) but was otherwise unremarkable. Viral panels were negative for hepatitis A, B, and C, HSV-1, HSV-2, and VZV. Antismooth muscle antibody and antimitochondrial antibody were negative. Ceruloplasmin was mildly elevated (35 mg/dL). Coagulation studies revealed an elevated prothrombin time (PT) of 14.5 seconds (international normalized ratio (INR) of 1.3), consistent with the known hepatic insult. Abdomen MRI revealed periportal and reactive gallbladder edema, consistent with acute hepatic inflammation, but there was no evidence of chronic liver disease or portal hypertension. Clinical evaluation focused on new symptoms suggestive of progressive liver injury, including jaundice, scleral icterus, nausea, vomiting, and abdominal pain, as well as complications from impaired liver function including edema, bleeding, and encephalopathy. By the time of admission, the patient's fever, fatigue, and chills had resolved. He remained asymptomatic throughout his hospital stay.

Based on the unremarkable workup, it was felt that the liver injury was primarily related to encorafenib/binimetinib, though simvastatin may have played a minor role. The concurrent kidney injury was thought to be multifactorial, with encorafenib/binimetinib, hydrochlorothiazide, and lisinopril all contributing to its development. The patient was discharged after four days with instructions to follow up with oncology and hepatology in the outpatient setting.

**Figure 1:**
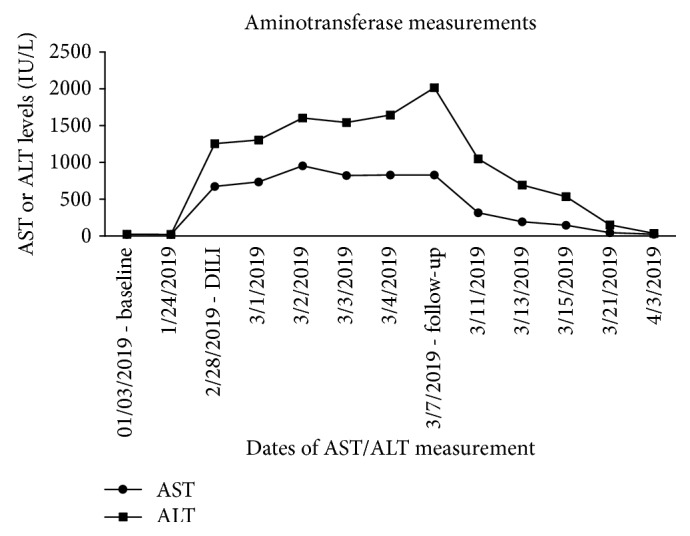
Aminotransferase measurements preceding, during, and following drug-induced liver injury. Measurements are expressed in international units per liter. AST peak was 950 on 03/02/2019; ALT peak was 2,007 on 03/07/2019. ALT: alanine aminotransferase; AST: aspartate aminotransferase; DILI: drug-induced liver injury.

The patient was seen in the outpatient oncology clinic three days after discharge, and updated labs were obtained. ALT was markedly elevated at 2,007 IU/L; AST was 825 IU/L. Total bilirubin and alkaline phosphatase were normal. Treatment was not reinitiated at this time, and serial CMPs were obtained every two to three days following. AST and ALT slowly returned to normal over the course of the next several weeks. No complications were noted during this timeframe.  Figure 1 summarizes the patient's AST/ALT trends from his baseline prior to the liver injury to his gradual return to normal limits a few weeks later. From a treatment standpoint, a subsequent positron emission tomography (PET)/computed tomography (CT) scan from April 2019 demonstrated tumoral response with decreased fluorodeoxyglucose (FDG) uptake in several previously noted soft tissue and bony metastases.

## 3. Discussion

For advanced melanoma, BRAFi and MEKi therapies are novel treatment options, which are rapidly becoming mainstays of treatment in select cases due to their rapid and robust tumoral response and generally well-tolerated AE profile [3, 4, 8, 9]. Nonetheless, as in our patient, severe AEs may be possible, necessitating hospital admission for workup and treatment [3, 4, 10]. Drug-induced liver injury (DILI) is one such complication.

Diagnosis of DILI is often difficult to confirm and may therefore be a diagnosis of exclusion, ruling out other causes such as liver metastases, viral infection, autoimmune disease, and ischemia [11]. Multiple assessment tools have been developed and validated in previous studies, but there is no consensus regarding their use in diagnosis of DILI [12]. As such, diagnosis often does not require the use of these scales for confirmation. In general, a clear temporal relationship between drug administration and liver injury, as well as exclusion of other causes, is the key finding to diagnose DILI [12]. Resolution following cessation of the drug further supports the conclusion. Biopsy is sometimes necessary if evaluations are equivocal. In our case, the patient's clinical picture was initially suggestive of DILI, and improvement of LFTs following drug discontinuation further substantiated this theory. Several alternative explanations were explored but were excluded after workup was found to be negative.

The manifestations of DILI can vary greatly, ranging from asymptomatic enzyme elevations to fulminant liver failure; consequently, several grading schemes have been developed to categorize DILI based on severity. Grades are assigned on a 5-point scale, with grade 1 representing mild disease and grade 5 representing fatal disease or need for transplant. Most assessments are stratified based on the degree of elevation noted in serum AST, ALT, alkaline phosphatase, gamma-glutamyl transferase, and total bilirubin levels [13, 14]. Abnormalities in PT/INR are also frequently employed in classification. Clinical findings involved in grading include length and severity of symptoms, including jaundice, pruritis, fatigue, weakness, nausea, anorexia, and weight loss [14]. Evidence of damage to another organ may also be used in classifying high grade DILI.

As it pertains to encorafenib/binimetinib, mild to moderate aminotransferase elevations were described in the COLUMBUS trial, but no grade 4 toxicity was reported [3]. The National Cancer Institute and Drug-Induced Liver Injury Network define grade 4 liver toxicity as aminotransferase elevations > 20x upper limit of normal [13] or acute liver injury resulting in other organ dysfunction (brain, kidney, etc.), respectively [14]. Our patient met both criteria, and to our knowledge, he represents the first reported case of grade 4 liver toxicity from combination encorafenib/binimetinib.

Regarding DILI management, the cornerstones of treatment are withdrawal of the offending agent and supportive care [12]. Close monitoring of AST, ALT, alkaline phosphatase, gamma-glutamyl transferase, total bilirubin, and PT/INR is necessary to monitor response. In most cases, DILI resolves without additional sequelae once the offending agent is removed, though it may take many weeks before laboratory studies normalize [14]. In our case, encorafenib/binimetinib was withdrawn the same day that the patient's AST/ALT levels were found to be first elevated. After several weeks, his laboratory studies normalized and no additional complications were noted. Given the severity of liver injury, we decided to permanently discontinue encorafenib/binimetinib.

Unrelated to the liver toxicity, an additional interesting aspect of this case is that even though the patient had eventually progressed on dabrafenib/trametinib (despite an overall impressive PFS of 20 months with this combination), he later had partial response to a different class of BRAFi/MEKi (encorafenib/binimetinib). This is to say that despite failing one line of BRAFi/MEKi therapies (along with radiation therapy and a short course of immune checkpoint inhibition), rechallenge with a different BRAFi/MEKi combination shortly afterward was at least partially successful, though the drugs' long-term utility was limited by the grade 4 DILI. We speculate that this outcome in our BRAFi-pretreated patient was due, in part, to a number of factors, such as BRAFi resensitization, immunotherapy exposure, and the pharmacologic profile of encorafenib.

Rechallenge with a different BRAFi and/or MEKi has been described previously as a possible therapeutic option for patients with melanoma who progress on a first BRAFi and subsequent second therapy from another drug class (such as checkpoint inhibitors) [15–17]. Interestingly, BRAFi-resistant melanoma cells become dependent on the inhibition for their growth, and consequently, withdrawal of the BRAFi leads to regression of the resistant cells [18]. The presence of a BRAFi-free period is therefore integral to the resensitization of the malignancy to BRAFi therapy. The relationship between duration of BRAFi holiday and tumor response rates has been explored previously, but the data thus far is conflicting, with some studies showing improved response rates with longer BRAFi-free intervals [17] and others showing no significant temporal correlation [15, 16]. The collective data is also unclear about whether AE profiles are affected by duration of BRAFi holiday, though one could speculate that this may be implicated. For our patient, the presence of the BRAFi holiday likely helped facilitate the partial response seen upon initiation of encorafenib/binimetinib.

There is suggestion that the therapy selected in the BRAFi-free period also plays a role in increasing tumor sensitivity on BRAFi rechallenge. In a small sample of patients, Roux et al. found improved responses to a second BRAFi if patients were treated with an immune checkpoint inhibitor during the BRAFi-free period [15]. However, these findings were not reproduced by Tietze et al. [16] or Valpione et al. [17]; therefore, it is unclear whether immunotherapy truly enhances tumor response on BRAFi rechallenge. Prospective data would be helpful to determine this relationship moving forward.

Encorafenib has a distinct pharmacologic profile compared to other BRAFi therapies, with a long dissociative half-life (greater than 30 hours), greater potency, and a stronger BRAF inhibitory effect [19]. It is also more selective than other BRAFi therapies for cells expressing the BRAFV600 mutation [20]. Underscoring this in the COLUMBUS study, the encorafenib/binimetinib combination was noted to have the longest PFS and OS among the available BRAFi and MEKi (with the caveat that this is an indirect comparison of different trials done at different times) [3]. These superior pharmacologic properties may help to explain the response to rechallenge in our patient.

## 4. Conclusion

Encorafenib and binimetinib represent newly approved BRAFi/MEKi therapies that have recently been employed in combination for the treatment of BRAFV600-mutant melanoma. The literature has shown that this combination offers a superior response and AE profile to other BRAFi monotherapies. That being said, care must still be taken to monitor for serious AEs from combination therapy. This case illustrates that rare serious AEs can be seen with novel cancer agents in the real-world setting. Discontinuation of the combination BRAFi/MEKi therapies led to successful reversal of liver injury. Regular clinical monitoring and LFT evaluation was essential in assessing response to management. This case adds to the available literature regarding hepatotoxicity with novel BRAFi and MEKi therapies and can help clinicians with management of such toxicities in the future. This case also highlights that there is a possible role for BRAFi/MEKi rechallenge to elicit clinical response.

## Abbreviations

AE: Adverse effect
ALT: Alanine aminotransferase
AST: Aspartate aminotransferase
BUN: Blood urea nitrogen
BRAFi: BRAF inhibitor
CBC: Complete blood count
CMP: Comprehensive metabolic panel
CT: Computed tomography
DILI: Drug-induced liver injury
FDG: Fluorodeoxyglucose
GI: Gastrointestinal
INR: International normalized ratio
LFTs: Liver function tests
MRI: Magnetic resonance imaging
MEKi: MEK inhibitor
OS: Overall survival
PET: Positron emission tomography
PFS: Progression-free survival
PT: Prothrombin time.
